# Withaferin-A kills cancer cells with and without telomerase: chemical, computational and experimental evidences

**DOI:** 10.1038/cddis.2017.33

**Published:** 2017-04-20

**Authors:** Yue Yu, Shashank P Katiyar, Durai Sundar, Zeenia Kaul, Eijiro Miyako, Zhenya Zhang, Sunil C Kaul, Roger R Reddel, Renu Wadhwa

**Affiliations:** 1 DAILAB, National Institute of Advanced Industrial Science & Technology (AIST), Tsukuba 305 8565, Japan; 2 Graduate School of Life & Environmental Sciences, University of Tsukuba, Tsukuba 305 8572, Japan; 3 DAILAB, Department of Biochemical Engineering & Biotechnology, Indian Institute of Technology (IIT) Delhi, New Delhi 110 016, India; 4 Cancer Research Unit, Children's Medical Research Institute, The University of Sydney, NSW 2145, Australia; 5 Department of Molecular Virology, Immunology & Medical Genetics, The Ohio State University, Ohio 43210, USA; 6 Nanomaterial Research Institute, AIST, Tsukuba 305 8565, Japan

## Abstract

Maintenance of telomere length is the most consistent attribute of cancer cells. Tightly connected to their capacity to overcome replicative mortality, it is achieved either by activation of telomerase or an Alternative mechanism of Lengthening of Telomeres (ALT). Disruption of either of these mechanisms has been shown to induce DNA damage signalling leading to senescence or apoptosis. Telomerase inhibitors are considered as potential anticancer drugs but are ineffective for ALT cancers (~15% of all cancers). Withaferin-A (Wi-A), a major constituent of the medicinal plant, *Withania somnifera* (Ashwagandha), has been shown to exert anti-tumour activity. However, its effect on either telomerase or ALT mechanisms has not been investigated. Here, by using isogenic cancer cells with/without telomerase, we found that Wi-A caused stronger cytotoxicity to ALT cells. It was associated with inhibition of ALT-associated promyelocytic leukemia nuclear bodies, an established marker of ALT. Comparative analyses of telomerase positive and ALT cells revealed that Wi-A caused stronger telomere dysfunction and upregulation of DNA damage response in ALT cells. Molecular computational and experimental analyses revealed that Wi-A led to Myc-Mad mediated transcriptional suppression of NBS-1, an MRN complex protein that is an essential component of the ALT mechanism. The results suggest that Wi-A could be a new candidate drug for ALT cancers.

Normal somatic cells have a finite life span that is regulated by tumour suppressor mechanisms and shortening of telomeres. Cancer cells circumvent telomere shortening by activation of telomerase, a ribonucleoprotein consisting of RNA (TR) and reverse transcriptase enzyme (TERT) component, which adds TTAGGG to telomeric ends. Ectopic expression of hTERT in normal human fibroblasts has been shown to induce elongation of telomeres, permanent cell proliferation and susceptibility to experimental transformation.^1^, ^2^ In contrast to the upregulation of telomerase in large majority of cancer cells, telomerase-negative cancer cells possess mechanisms referred to as ALT (Alternate Lengthening of Telomeres).^3^ ALT cells are characterized by very heterogeneous telomeres and possess large nuclear structures (ALT-associated Promyelocytic Leukemia (PML) Body) called APB that contain telomeric DNA and several proteins including PML, TRF1, TRF2, Replication factor A, RAD51 and RAD52.^4^, ^5^, ^6^, ^7^ Reconstitution of telomerase activity in ALT cells has revealed that the human cells are capable of utilizing telomerase-dependent and -independent mechanisms of telomere maintenance concomitantly.^8^, ^9^ ALT has been detected not only in cultured cancer cells but also in tumour tissues accounting for 10–15% of all cancers, with high prevalence (≥ 20%) of liposarcoma, epithelioid sarcoma, chondrosarcoma, astrocytoma, malignant fibrous histiocytoma, glioblastoma multiforme, gastric carcinoma and neuroblastoma.^10^, ^11^, ^12^

The MRE11-RAD50-NBS1 (MRN) protein complex acts as a DNA damage sensor and controls DNA repair, cell cycle, telomere maintenance and genome stability by regulation of ATM (ataxia-telangiectasia mutated), ATR (ataxia-telangiectasia and Rad-3-related) and DNA PKcs (DNA protein kinase catalytic subunit) activities.^13^, ^14^, ^15^ MRN is essential for timely activation of ATM-mediated pathways and its dysfunction causes genome instability and premature ageing disorders, including ataxia telangiectasia (A-T), A-T-like disorder (ATLD) and Nijmegen breakage syndrome (NBS).^16^, ^17^, ^18^ Overexpression of NBS1 protein was shown to increase cell proliferation,^19^ its knockdown led to hypermutability and telomere changes that have been related to cancer predisposition.^20^ Hypomorphic mutations of the MRE11 gene lead to ATLD.^21^ Cells compromised for RAD50 also showed rapid shortening of chromosome ends and end-to-end chromosome fusions.^22^, ^23^ siRNA-mediated depletion of any subunits of MRN complex led to depletion of other subunits of the complex suggesting their co-regulation.^14^ MRN is found in APBs, and overexpression of Sp100, which caused sequestration of MRN proteins away from APBs, resulted in repression of the ALT mechanism, which was manifested by telomere length changes and suppression of APB formation,^24^ suggesting that MRN is involved in ALT. MRN complex proteins are regulated by c-Myc and n-Myc cellular oncogenes.^25^ Because of high incidence of telomerase activation in a wide variety of cancers, anti-telomerase drugs are considered useful for therapy. However, ALT tumours will be refractory to such drugs, so identification and characterization of new anti-ALT molecules is essential.

Withaferin-A (Wi-A) is a steroidal lactone found in the medicinal plant, *Withania somnifera* (Ashwagandha). It has anticancer activity attributable to (i) cell cycle arrest by downregulation of cyclin B1, cyclin A, Cdk2 and p-Cdc2 expression and increase in the levels of p-Chk1 and p-Chk2, (ii) downregulation of HPV E6 and E7 oncoproteins, (iii) induction and accumulation of p53, (iv) increased levels of p21^WAF1^, (v) a decrease in the levels of STAT3, (vi) an increase in p53-mediated apoptotic markers-Bcl2, Bax, caspase-3, cleaved PARP and Par-4, (vii) downregulation of AKT and EMT signalling and (viii) disruption of cytoskeleton elements including actin, vimentin and intermediate filaments suggesting that it is a potential natural anticancer drug.^26^, ^27^, ^28^, ^29^ In the present study, we investigated the effect of Wi-A on isogenic telomerase-plus (TEP) and -minus (ALT) cancer cells and found that it causes a stronger cytotoxicity to ALT cells. We provide experimental and computational evidences that Wi-A causes apoptosis in ALT cells by inhibition of ALT phenotype, induction of telomere dysfunction and Myc-Mad mediated transcriptional suppression of NBS-1, an MRN complex protein, an essential component of ALT mechanism.

## Results

### Wi-A caused stronger cytotoxicity to ALT cells

**Figure 1:**
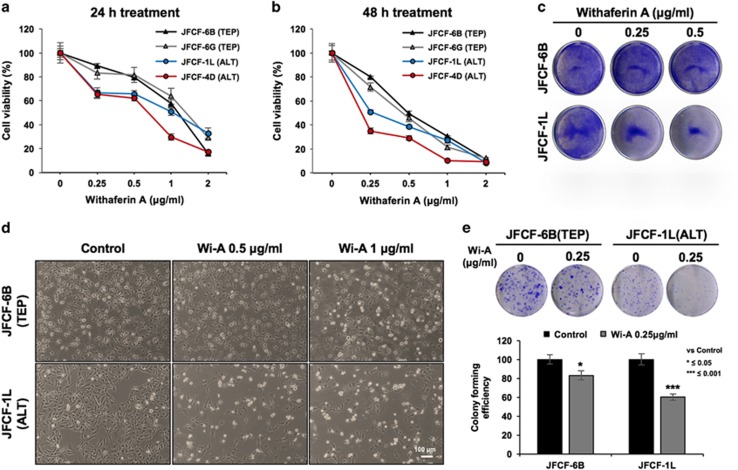
ALT cells are sensitive to Withaferin-A (Wi-A). (**a**) Cell viability assays of human telomerase-plus (TEP) and -minus cells possessing Alternate mechanism of Telomere Lengthening (ALT) showing dose-dependent cytotoxicity. ALT cells showed stronger toxicity as compared to the TEP cells both at 24 h (**a**) and 48 (**b**). Crystal Violet staining in control and Wi-A treated cells showing stronger response of ALT cells to Wi-A treatment (**c**). Phase contrast images of control and Wi-A treated TEP and ALT cells; stronger cytotoxicity and apoptosis in ALT cells were observed (**d**). Long-term viability assay by colony forming efficiency of control, Wi-A treated TEP and ALT cells showed stronger response of ALT cells to the treatment (**e**). Quantitation from three independent experiments is shown. *P<0.05 and ***P<0.001 denote statistical significance

We used two pairs of isogenic cells with and without telomerase, to determine the cytotoxicity in response to Wi-A. As shown in Figures 1A−C, a dose-dependent cytotoxic response was observed in all four cell lines (Telomerase Plus, TEP and telomerase negative, ALT). Of note, ALT (JFCF-1 L and JFCF-4D) cells showed stronger cytotoxicity at all doses as compared to the Telomerase Plus (TEP; JFCF-6B and JFCF-6G) cells. The IC_50_ values (μg/ml) for 24 and 48 h treatments were 0.6 and 0.19 (JFCF-4D) and 0.9 and 0.25 (JFCF-1 L) for ALT, and 1.2 and 0.44 (JFCF-6B) and 1.4 and 0.5 (JFCF-6G) for TEP cells, respectively. The results were confirmed by visual observations; 0.5 and 1 μg/ml of Wi-A caused apoptosis in ALT, but not TEP, cells (Figure 1d). Colony forming assays with low dose (0.25 μg/ml) of Wi-A also revealed its stronger cytotoxicity to ALT cells (Figure 1e). Furthermore, tumour-derived ALT (U2OS; osteosarcoma) cells also showed higher cytotoxicity than TEP (MCF7; breast carcinoma) cells (Supplementary Figure S1A). We investigated the molecular mechanism of its differential effect in response to low dose of Wi-A on TEP and ALT cells.

### Low dose of Wi-A induced apoptosis in ALT cells

**Figure 2:**
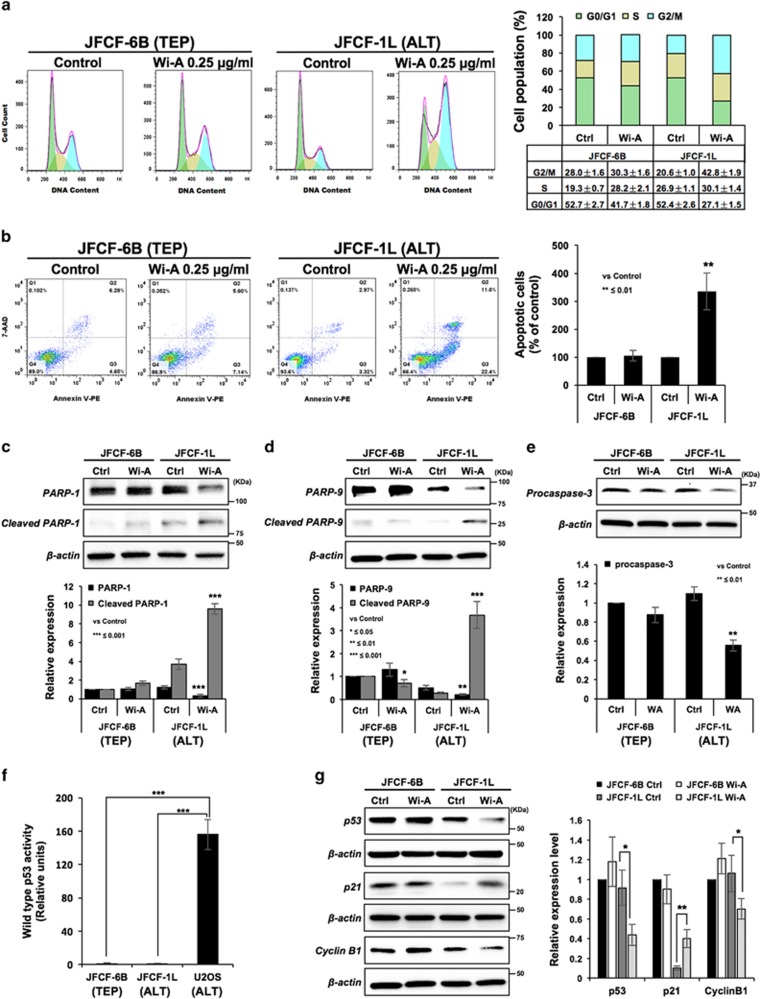
Wi-A caused G2/M arrest and apoptosis in ALT cells. (**a**) Cell cycle analysis showing increase in number of ALT cells at G2 arrest with Wi-A treatment. TEP cells did not show similar increase at equivalent dose. (**b**) Flow cytometric analysis showing increased apoptotic cell population with Wi-A treatment in ALT, but not TEP, cells. Quantitation from three independent experiments is shown on the right. (**c, d**) Western blotting analysis for molecular markers for apoptosis in control and Wi-A treated TEP and ALT cells. The data show cleavage of PARP-1 (**c**) and PARP-9 (**d**) in ALT, but not in TEP, cells treated with Wi-A. (**e**) Decrease in Procaspase-3 in Wi-A treated ALT cells. (**f**) p53-dependent luciferase reporter assay showing lack of p53 wild type activity in TEP and ALT cells. U2OS cells were used as a control for wild type p53. (**g**) Western blot analysis of control and Wi-A treated TEP and ALT cells showing decrease in p53, increase in p21 and decrease in Cyclin B in Wi-A treated ALT, but not TEP, cells. Quantitation from three independent experiments is shown on the right. *P<0.05, **P<0.01 and ***P<0.001 denote statistical significance

Cell cycle analysis of control and Wi-A treated cells showed a significant increase in the number of JFCF-1L (ALT), but not JFCF-6B (TEP), cells in G_2_/M phase (Figure 2a). Furthermore, as determined by Annexin V staining, Wi-A treated ALT, but not TEP, cells showed strong induction of apoptosis (Figure 2b). Western blotting of proteins critical for apoptosis and G_2_/M transition, we found a marked increase in the cleaved PARP-1 and PARP-9 (established makers of apoptosis) in Wi-A treated ALT, but not TEP, cells (Figures 2c and d); total PARP-1/9 showed decrease. Furthermore, procaspase-3 decreased in Wi-A treated ALT cells, confirming apoptosis (Figure 2e).

We next examined the expression of p53, p21 and Cyclin B1, the key regulators of cell cycle progression. As shown in Figure 2f, both ALT and TEP cells did not show wild type p53 activity as determined by reporter assay. Western blotting for p53 revealed its downregulation in Wi-A treated ALT cells only (Figure 2g). The latter also showed increase in p21 and decrease in Cyclin B1 (Figure 2g). Taken together, these data suggested that stronger toxicity of Wi-A to ALT cells is mediated by induction of G2/M arrest and apoptotic signalling.

### Wi-A caused inhibition of ALT phenotype

**Figure 3:**
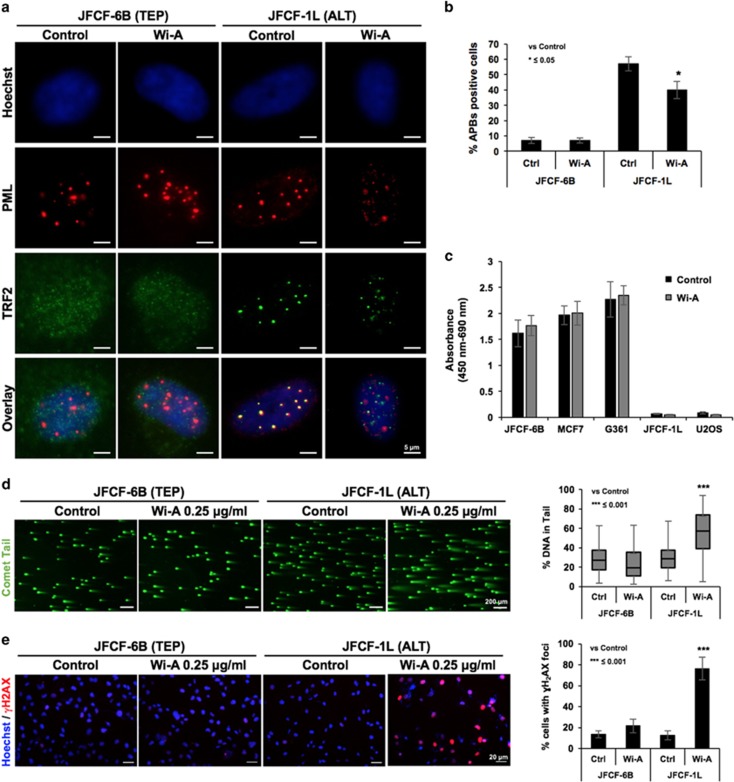
Wi-A caused disruption of ALT phenotype. (**a**) Co-immunostaining of TRF2 and PML (APB bodies) in control and Wi-A treated TEP and ALT cells showed APB bodies only in control ALT, but not in control TEP or in either of treated, cells. Disruption of APBs was observed in Wi-A treated cells. (**b**) Quantitation of APB bodies in control and Wi-A treated TEP and ALT cells from four independent experiments. (**c**) TRAP assay in control and Wi-A treated cells showed no effect of Wi-A on telomerase activity. (**d**) Neutral comet assay in control and Wi-A treated TEP and ALT cells showing increase in DNA damage in treated ALT cells only. Quantitation from three independent experiments is shown on the right. (**e**) Immunostaining of *γ*H2AX in control and Wi-A treated TEP and ALT cells showing increase in the number of *γ*H2AX positive cells in Wi-A treated ALT cells. Quantitation from at least 200 cells is shown on the right. *P<0.05 and ***P<0.001 denote statistical significance

In order to get molecular insights to the stronger cytotoxicity of Wi-A to ALT cells, we examined a well-established marker for ALT, APBs (ALT-associated PML nuclear bodies). As expected, APBs (detected here by co-localization of the TRF2 telomeric binding protein and PML protein) occurred specifically in ALT cells (Figure 3a). Of note, Wi-A treated ALT cells showed 20−40% reduction in APBs (Figure 3b). On the other hand, telomerase activity (determined by TRAP assay) in control as well as Wi-A treated TEP (JFCF-6B, MCF-7 and G361) and ALT (JFCF-1L and U2OS) cells remained unchanged (Figure 3c).

Wi-A has been shown to induce DNA damage and oxidative stress response in cancer cells.^30^, ^31^ We hence anticipated that the high sensitivity of ALT cells to Wi-A may be due to stronger induction of DNA damage response. Neutral comet assay to examine DNA double strand breaks (DSB) directly showed a marked (twofold) increase in DSB formation in Wi-A treated ALT cells (Figure 3d). TEP cells treated with equivalent dose (0.25 μg/ml) of Wi-A showed no difference in DSB content with respect to the control group (Figure 3d) suggesting that these cells are somewhat resistant to Wi-A induced DSB. The induction of DNA breaks after Wi-A treatment was also examined by *γ*H2AX immunostaining. Whereas TEP cells showed no difference in *γ*H2AX staining between control and Wi-A (0.25 μg/ml) treated cells, JFCF-1L cells showed a large increase in the number of *γ*H2AX positive cells (Figure 3e). These data were consistent with Comet assay results and suggested that Wi-A caused stronger DNA damage response in ALT cells.

### Wi-A caused telomere dysfunction in ALT cells

**Figure 4:**
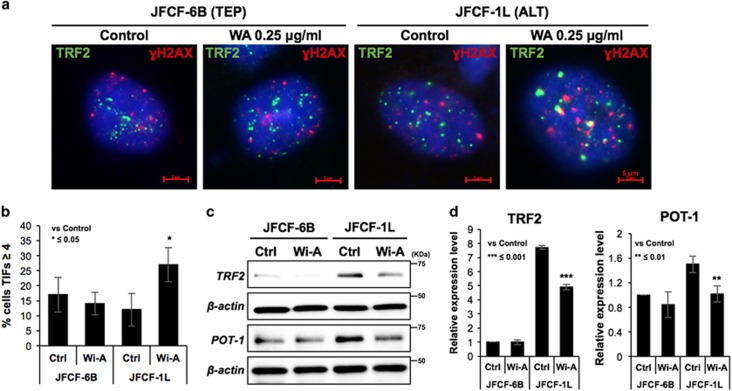
Wi-A caused telomere dysfunction. (**a**) Co-immunostaining of TRF2 and *γ*H2AX in control and Wi-A treated TEP and ALT cells showing telomere dysfunction in ALT cells. Quantitation of the data is shown in (**b**). (**c**) Western blotting of TRF2 and POT-1 in control and Wi-A treated TEP and ALT cells showing decrease in their expression in ALT, but not TEP, cells. β-actin was used as an internal control in all gels. Since PARP-9 and Cyclin B1 (Fig. 2) and TRF2 (**c**) were detected on the same blot, β-actin is same. (**d**) Quantitation from three independent experiments is shown on the right. *P<0.05, **P<0.01 and ***P<0.001 denote statistical significance

In order to investigate whether treatment with Wi-A affects telomere length, we performed qPCR-based telomere length assays in control and Wi-A treated TEP and ALT cells. Both the cell lines did not show any effect of Wi-A on telomere length (Supplementary Figure S2) suggesting that it may alter telomere structure or cause telomere damage/de-protection. We hence performed quantitative assay for telomere dysfunction in control and Wi-A treated cells. The number of cells with telomere-dysfunction-induced foci (TIF) as examined by co-localization of *γ*H2AX and TRF2 signals (Figure 4a) showed significant increase (TIFs ≥4; ~10% to ~25%) in ALT cells (Figure 4b). In contrast, TEP cells showed no noticeable difference in telomeric DNA damage (Figures 4a and b).

Several studies have provided evidence that the telomere binding proteins TRF2 and POT1 are predominantly involved in chromosome end protection by preventing activation of a DNA damage response.^32^, ^33^ We next considered whether Wi-A affects TRF2 and POT1. Western blotting revealed downregulation of both these proteins in Wi-A treated ALT cells; TEP cells possessed a low level of expression that was not altered by Wi-A treatment (Figures 4c and d). These data suggested that stronger cytotoxicity of Wi-A to ALT cells is due to, at least in part, its effect on telomere protecting proteins.

### Wi- A caused downregulation of MRN complex proteins in ALT cells

**Figure 5:**
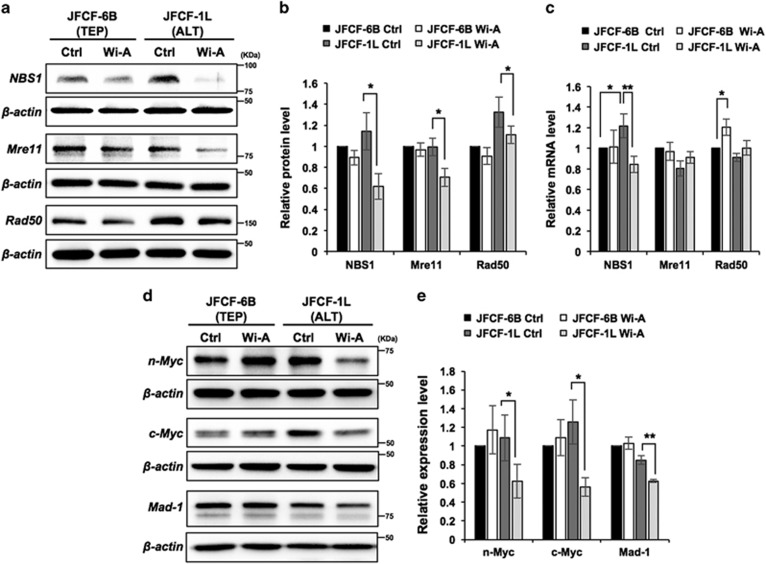
Wi-A caused downregulation of MRN complex proteins. (**a**) Western blotting of MRN complex proteins (NBS1, Mre11 and Rad50) in control and Wi-A treated TEP and ALT cells showing significant decrease in their expression in ALT cells. (**b**) Quantitation from three independent experiments is shown. (**c**) Real-time qRT-PCR analysis showing decrease in NBS1 expression in Wi-A treated ALT cells was most pronounced and significant. (**d**) Western blotting of n-Myc, c-Myc and Mad-1 (upstream transcriptional regulators of MRN complex proteins) in control and Wi-A treated TEP and ALT cells showing stronger decrease in their expression in the latter. (**e**) Quantitation from three independent experiments is shown on the right. *P<0.05 and **P<0.01 denote statistical significance

Based on the above data that Wi-A targets ALT mechanism, we examined the status of MRN complex proteins; essential for the ALT mechanism and APB assembly.^34^, ^35^ Western blotting revealed a sharp decline in NBS1 and MRE11 expression and a smaller reduction of RAD50 in Wi-A treated ALT cells. TEP cells showed only a small/negligible change (Figures 5a and b). Of note, tumour-derived ALT (U2OS) cells showed high level of NBS1 protein expression that gets attenuated in response to Wi-A treatment; TEP (MCF7) cells did not show such effect (Supplementary Figure S1B). Real-time PCR quantitation of their transcripts showed reduction in NBS1, but not in MRE11 and RAD50, in ALT cells. Rather, a slight increase was observed in MRE11 and RAD50 in response to treatment with Wi-A. TEP cells also showed a small increase in RAD50 (Figure 5c). Since MRN complex is regulated by n-Myc and c-Myc at the transcription level,^19^, ^25^ we next performed western blotting to examine their expression in control and Wi-A treated ALT and TEP cells. Of note, treatment with Wi-A led to ~40% decrease in n-Myc and ~55% decrease in c-Myc in JFCF-1L (ALT) cells. However, a slight increase of these two proteins was observed in JFCF-6B (TEP) cells after the treatment (Figure 5d).

The transcriptional activation function of Myc proteins is regulated by their heterodimer complex formation with Max, and the antagonistic repressor Mad-Max (the heterodimer that competes with Myc-Max complex to bind to the identical genomic sites and, consequently, inhibits transactivation mediated by Myc-Max heterodimer). We next examined Mad-1 in ALT and TEP cells following treatment with Wi-A. Whereas the treatment did not alter Mad-1 expression in TEP cells, there was a small decrease in ALT cells (Figure 5e). Of note, Wi-A induced reduction in n-Myc and c-Myc proteins (about 50%) was greater than that of Mad-1 (about 25%), suggesting that the Wi-A treated cells possess more of Mad-1-Max than the Myc-Max complex resulting in transcriptional repression of the downstream effectors (such as NBS1) as supported by data shown in Figure 5c.

### Molecular dynamic and computational insights to the effect of Wi-A on DNA/ Myc-Max and /Mad-Max complexes

We first investigated the DNA binding affinity of Myc-Max and Mad-Max proteins. Although Myc and Mad dimerize with the same Max protein (to form Myc-Max/Mad-Max) in order to bind with DNA E-box via its bHLHZ domain,^36^ the identity between the Myc and Mad sequences was below 28% when calculated using BLOSUM90 scoring matrix and word size 2 of protein BLAST. It was hypothesized that difference of residues in the DNA-binding site of Myc-Max and Mad-Max should lead to different binding affinities with the DNA. We tested this prediction by docking proteins with the DNA. HADDOCK docking score of Myc-Max with DNA was −190.6 while docking score of Mad-Max with the DNA was −275.1 (Table 1); suggesting that the Mad-Max protein complex had a significantly higher binding affinity than Myc-Max protein complex towards DNA. Higher affinity of Mad-Max towards DNA accounted for the antagonistic nature of Mad protein in competition with Myc protein.

**Figure 6:**
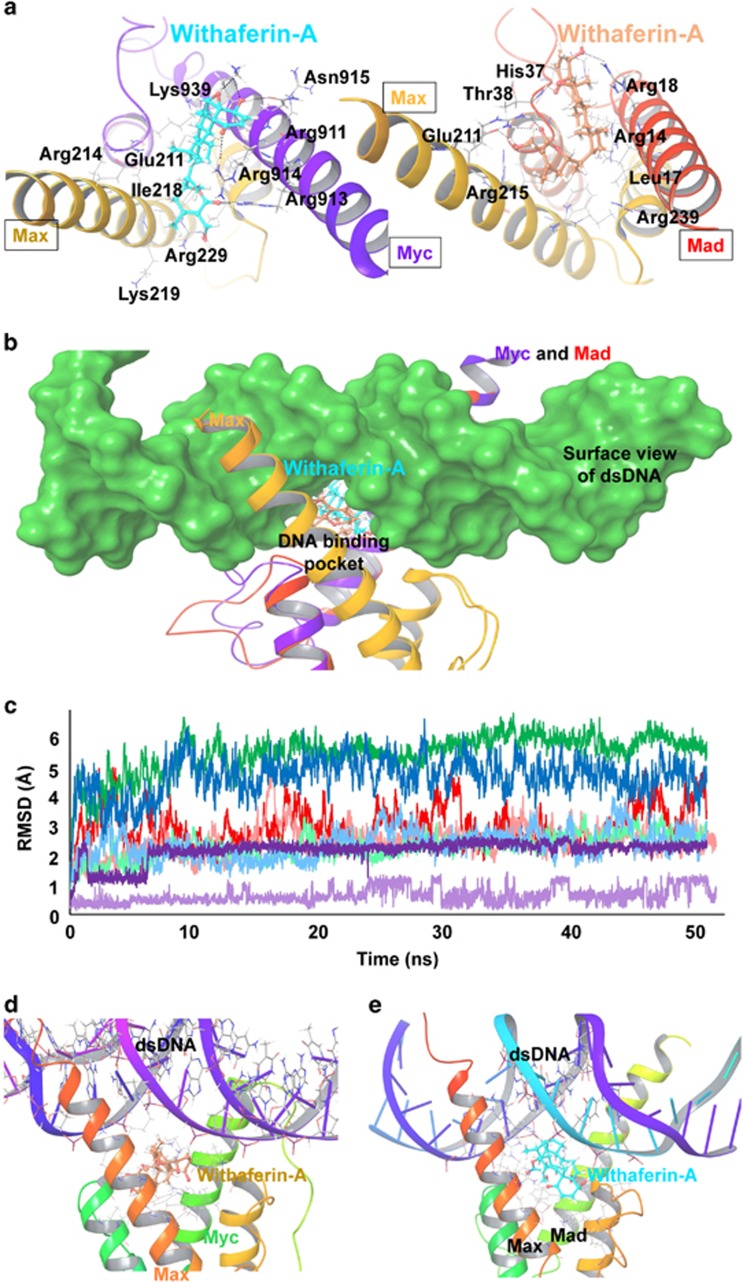
Binding of Wi-A with Myc-Max and Mad-Max complexes and its effect on structural stability. (**a**) Interactions of Wi-A with Myc-Max (left) and Mad-Max protein complexes after the molecular docking. Wi-A binds at the bottom of DNA-binding site in both types of protein complexes. Docking poses of Wi-A were interacting with Myc-Max protein complex via residues Arg911, Arg913, Arg914, Asn915, Leu917 of Myc and via residues Glu211, Arg214, Arg215, Lys219, Phe222 and Arg239 of Max protein. Interacting residues of Mad-Max complex with Wi-A were Arg13, Arg14, Leu17, Arg18, His37, Thr38 of Mad, and Glu211, Arg214, Arg215, Phe222, and Arg239 of Max protein. (**b**) Superimposition of Wi-A docked proteins with DNA bound forms to inspect the clash between Wi-A and DNA binding site. In both Myc-Max and Mad-Max, Wi-A takes place at the empty space between arms of Myc/Mad and Max proteins, without interfering with DNA binding. (**c**) RMSD fluctuations of Myc-Max protein backbone (red) and Mad-Max protein backbone (light red) during 50 ns simulation of Wi-A bound protein complexes. RMSD fluctuations of Myc-Max protein backbone (green) and Mad-Max protein backbone (light green) during 50 ns simulation of Wi-A bound DNA-protein complexes. RMSD fluctuations of DNA in Wi-A bound Myc-Max-DNA complex (blue) and Mad-Max-DNA complex (light blue) during 50 ns simulation. RMSD fluctuations of all three DNA, protein and ligand in Wi-A bound Myc-Max-DNA complex (purple) and Mad-Max-DNA complex (light purple) during 50 ns simulation. (**d**) Depiction of stabilized Wi-A in DNA-bound bound Myc-Max after 50 ns molecular dynamics simulation. (**e**) Depiction of stabilized Wi-A in DNA-bound Mad-Max proteins after 50 ns molecular dynamics simulations

We next examined the effect of Wi-A binding on Myc-Max-DNA and Mad-Max-DNA complexes. Binding site analysis revealed that the bHLHZ domains of Myc and Mad proteins possess a potential ligand-binding site. Hence, residues of DNA binding sites in both Myc-Max and Mad-Max protein complexes were chosen as the binding site for Wi-A. Docking poses of Wi-A revealed −5.47 kcal/mol and −5.86 kcal/mol docking scores with Myc-Max and Mad-Max after the energy minimization (Table 1). Upon superimposition of DNA over the docked pose of Wi-A with Myc and Mad, it was found that Wi-A was not interfering with the DNA nucleotides, neither in Myc-DNA complex nor in Mad-DNA complex, though the residues were within the DNA binding region, (Figures 6a and b). These results suggest that Wi-A is able to bind at the DNA-binding site of Myc and Mad and does not hinder the binding of these transcription factors to DNA.

Stability of Wi-A at the DNA binding site of Myc and Mad proteins was revealed during the 50 ns long molecular dynamics simulations. Root mean square deviation (RMSD) values of Wi-A were found highly stable throughout the simulation with Myc as well as with Mad. Simulation analysis showed that (i) Wi-A made highly stable complex with Mad and (ii) it also stabilized the Mad-Max complex more than Myc-Max complex (Figure 6c). Local interactions around Wi-A were much stronger in Mad as compared to Myc, which explains high stabilization of Mad-Max protein complex as compared to Myc-Max protein complex.

Wi-A stabilized Myc-Max and Mad-Max protein complexes were further used to check the possibility of Wi-A occupying the DNA-binding space. For this purpose, DNA from the crystal structure was superimposed over one of the snapshots (protein structure) of stable trajectory of 50 ns simulation to create protein-DNA-ligand complexes (complex between DNA, Wi-A and Myc-Max/Mad-Max). As suggested from the docking studies, even within stabilized protein and Wi-A, it was found that Wi-A did not interfere with any of the nucleotides of DNA. These results suggest that Wi-A is able to stably bind at the bHLHZ domain of Myc and Mad without interfering with DNA binding. Further molecular dynamics simulations were conducted to study the effect of Wi-A binding on DNA binding affinity using the superimposed protein-DNA-ligand complexes. Though backbone of both Myc and Mad proteins were stable and did not fluctuate much during the 50 ns molecular dynamics simulation, backbone of Mad was comparatively more stable, showing deviation below 3.5 Å as opposed to 5 Å of Myc-DNA complex (Figure 6c). DNA was binding with high stability with Myc-Max and Mad-Max after the binding of Wi-A during 50 ns simulations. Even in the presence of DNA, Wi-A stably occupied the space in DNA-binding site without interfering with DNA-binding in the case of both Myc-Max and Mad-Max complexes (Figures 6d and e). Analysis of hydrogen bonds revealed that, after the binding of Wi-A, the number of hydrogen bonds increased during the simulations of both Myc-Max and Mad-Max proteins (Supplementary Figure S3). These data suggested that Wi-A actually increases the binding affinity between the Myc-Max/Mad-Max proteins and DNA. To further confirm this, we analysed the total binding interface area between proteins and DNA, before and after the binding of Wi-A. After the binding of Wi-A, the binding interface between protein and DNA increased both in Myc-Max/DNA and Mad-Max/DNA complexes (Table 1). Further, the RMSD of protein-ligand-DNA complexes suggested the existence of stable complex between proteins, DNA and Wi-A.

The HADDOCK score of Mad-Max protein and DNA was calculated to be much higher than Myc-Max and DNA, which indicated the stronger binding of Mad with DNA as compared to Myc. Relatively higher affinity of Mad towards DNA enhanced further after the binding of Wi-A. For the Wi-A -bound Mad-DNA complex, the increase in number of hydrogen bonds as well as the binding interface between protein and DNA was higher when compared to the Myc-DNA complex. These data suggested that Wi-A promotes the binding of Mad with DNA more than the binding of Myc, thereby enhancing the repressor function of Mad-Max complex against the activation of Myc-Max complex.

**Figure 7:**
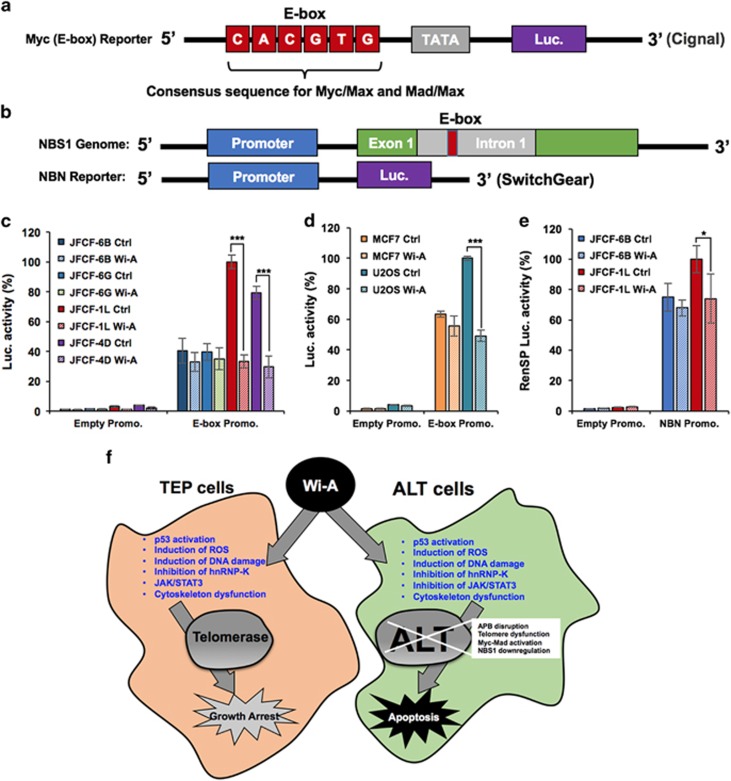
Wi-A caused repression of NBS-1 in ALT cells. Schematic presentation of reporters, E-box (Cignal) reporter containing Myc/Mad binding consensus sequence (**a**) and NBS-1 promoter (SwitchGear) lacking the Myc/Mad binding site (**b**) are shown. (**c**) E-box reporter assays in control and Wi-A treated TEP and ALT cells showed ~70% decrease in ALT, but not in TEP, cells. (**d**) Tumour-derived ALT (U2OS), but not the TEP (MCF7), cells also showed decrease in E-box driven luciferase reporter in Wi-A treated cells. (**e**) NBS-1 promoter reporter assay on control and Wi-A treated TEP and ALT cells did not show equivalent decrease in Wi-A treated cells. Quantitation from three independent experiments is shown. (**f**) A model showing the effect of Wi-A on TEP and ALT cells. It targets several cancer promoting proteins and mechanism in these cells (shown in blue). In addition, several ALT cell-specific mechanisms (APB bodies, downregulation of NBS1 through Myc-Mad activation and induction of telomere dysfunction) are affected causing their apoptosis. ***P<0.001 denotes statistical significance

We next performed NBS1-promoter luciferase assay in control and Wi-A treated cells. Two kinds of luciferase reporters, (i) NBS1 promoter and (ii) E-box containing Myc/Mad binding consensus sequences (Figures 7a and b) were used to determine the effect of Wi-A on Myc/Mad-driven transcriptional regulation. As shown in Figure 7c, we found that Wi-A caused strong suppression (~70%) of the E-box driven luciferase reporter in two ALT cell lines. Of note, the same reporter showed only ~10% decrease in Wi-A treated TEP cell lines. Similar results were obtained in tumour-derived cells (Figure 7d) and were also supported by decreased levels of NBS1 protein and mRNA in Wi-A treated ALT cells (Figures 5a−c). Furthermore, effect of Wi-A was compromised in case of NBN reporter that lacked Myc/Mad binding sites (Figures 7b and e) suggesting that Wi-A mediated transcriptional suppression was mediated predominantly by binding to the Myc/Mad-sites and supported the above computational and molecular dynamic analyses.

## Discussion

Telomere shortening evokes a DNA damage response that initiates senescence and apoptosis to avoid tumorigenesis.^2^, ^37^ Cancer cells escape from telomere shortening by mechanism(s) involving either telomerase or ALT.^10^, ^38^, ^39^ Both of these are considered as prime drug targets for cancer therapy. More efforts have been focused on the telomerase inhibitors due to high incidence (80–90%) of telomerase activation as compared to ALT (10–20%), which is most prevalent in tumours such as astrocytomas, glioblastomas, osteosarcomas, fibrous histiocytomas and liposarcomas.^11^, ^40^ Recently, an ATR inhibitor, VE-821 was shown to cause stronger DNA damage to ALT cancer cells.^41^ *In vitro* studies have demonstrated that telomerase and ALT mechanism can coexist within individual cancer cells.^9^ Inhibition of TEP could activate ALT or *vice versa*^42^, ^43^ raising the need for dual action inhibitors. We report that Wi-A is a good candidate for cancer treatment. It caused strong cytotoxicity to ALT cells (Figures 1 and 2). Although no effect on telomere length (Supplementary Figure S2) and telomerase activity (Figure 3c) was observed, it kills TEP cells by telomerase-independent mechanisms as reported previously.^30^, ^31^, ^44^ We demonstrated that Wi-A caused strong telomere damage in ALT cells by suppression of shelterin proteins (TRF2 and POT1) (Figure 4). Furthermore, it caused downregulation of Myc and the MRN complex protein-NBS1, an essential protein for ALT mechanism and cell survival.^19^, ^24^, ^25^ Zhong *et al* have shown that the knockdown of NBS1 resulted in inhibition of ALT-mediated telomere maintenance, decreased numbers of ALT-associated PML bodies and decreased telomere length.^34^

We, for the first time, demonstrate with the help of molecular docking, computational and experimental approaches that Wi-A inhibits Myc. Extensive molecular docking analysis of Wi-A and Myc-Max/Mad-Max complexes revealed that (i) Wi-A promotes the binding of Mad with DNA more than Myc, thereby enhancing the antagonistic function of Mad-Max complex against the Myc-Max complex and (ii) Wi-A stabilized the Mad-Max complex, notably more than Myc-Max complex, resulting in its stronger binding affinity with DNA. By protein, mRNA and reporter assays, we found that the Wi-A treated ALT cells experience strong suppression of NBS1 protein that was predominantly mediated by Mad/Max (Figures 5a−c and 7a). Taken together, we report that in addition to several common targets in TEP and ALT cancer cells (Figure 7f, shown in blue), Wi-A targets ALT specific proteins involved in maintenance of telomere length and hence it is considered a good candidate. Although further studies are warranted to resolve yet other molecular targets/mechanisms and pharmokinetics related to stronger toxicity of Wi-A in ALT cells and tumours, it is likely that Myc/Mad-driven suppression of NBS1 plays a major role in this phenomenon for the reasons (i) NBS1 is upregulated in ALT cells and is an essential survival factor for these cells.^24^, ^34^ TEP cells, although equally proliferative and malignant, may depend less on NBS1 function and (ii) transcription factor Myc (upregulated in ALT cells), and its antagonist Mad together regulate NBS1 expression; Wi-A caused downregulation of c-Myc and n-Myc. It was also predicted to increase the binding of Mad to DNA. Collectively, these effects translated to decrease the expression of NBS1 in ALT cells; well supported by mRNA as well as protein analysis. Continued extensive studies may further highlight the value of Wi-A as an effective anticancer natural drug.

## Materials and methods

### Preparation of Withaferin-A

Withaferin-A (Wi-A) was prepared from dried Ashwagandha leaves, as described previously.^44^ A stock of Wi-A (10 mg/ml; 20 mM) was prepared in DMSO and stored in −20 ^o^C. Working concentrations of Wi-A were prepared in cell culture medium.

### Cell culture, treatments, proliferation and cell cycle assays

Human breast carcinoma (MCF-7), melanoma (G361) and osteosarcoma (U2OS) were obtained from Japanese Collection of Research Bioresources (JCRB, Japan). The cells were authenticated by the source. Cells were frozen in −80 ^o^C and LN_2_ in multiple vials and were cultured (described below) for less than 50 population doublings for the current study. SV40-immortalized human TEP and ALT fibroblast lines were generated from the same parental donor cell (JFCF-6), and the details are described in Supplementary Table 1. Cells were cultured in Dulbecco's modified Eagle's medium (DMEM; Gibco BRL, Grand Island, NY, USA) and treated with Wi-A at about 60% confluency.^44^ Morphological observations, crystal violet staining and cell viability (MTT and colony-forming assays) were determined as described.^45^ Cell cycle and apoptosis analyses were performed using EasyCyte Guava cytometer (Millipore, Billerica, MA, USA) and FlowJo software (version 7.6, Flow Jo, LLC, USA) as described earlier.^45^ Apoptotic cells were detected by Guava Nexin Reagent, a pre-made cocktail containing Annexin V-PE and 7-AAD.^45^

### Antibodies

Rabbit anti-PARP-1 (H-250), anti-caspase-3 (H-277) and anti-POT1 (H-200) (Santa Cruz, CA, USA); goat anti-PML (N-19) (Santa Cruz); mouse anti-p53 (DO-1), anti-Cyclin B1 (GNS1), anti-N-myc (B8.4.B) and anti-TRF2 (4A794) (Santa Cruz); rabbit anti-PARP-9, anti-PML, anti-c-Myc (Y69); mouse anti-Rad50 (13B3/2C6), anti-Mre11 (12D7) and anti-NBS1 (NBS1-501) (Abcam, Cambridge, MA, USA); rabbit anti-phospho-histone H2A.X (20E3), anti-p21 (12D1) and anti-Rad50 (Cell Signaling, MA, USA); mouse anti-TRF2 (Cell Signaling); mouse anti-phospho-histone H2A.X (Millipore); rabbit anti-MAD1 (Gene Tex, CA, USA), rabbit anti-NBS1(Novus, Cambridge, UK) and anti-beta actin (AC-15) (Abcam) antibodies were used.

### Luciferase reporter assay

PG13-luc (wt p53 binding sites), a gift from Bert Vogelstein, and RenSP luciferase reporter construct containing the promoter region of NBS1 (SwitchGear Genomics (Menlo Park, CA, USA), and Myc-responsive luciferase construct encoding tandem repeats of E-box sequence (Cignal Myc Reporter kit, Qiagen, Standford Valencia, CA, USA) were transfected using either Lipofectamine 2000 (Invitrogen, Carlsbad, CA, USA) or XtremeGENE HP (Roche, Basel, Switzerland). Luciferase activity was detected using either a dual luciferase reporter assay system (Promega, Madison, WI, USA) or LightSwitch Dual Assay Systems (SwitchGear Genomics) following the manufacturer's protocol (also detailed in Supplementary Information).

### Telomerase activity detection (TRAP assay)

Telomerase activity was determined with a PCR-based telomeric repeat amplification protocol (TRAP) enzyme-linked immunosorbent assay (ELISA) kit (Roche, Mannheim, Germany) following the manufacturer's protocol (also detailed in Supplementary Information).

### Comet assay

DNA double-strand breaks were quantitated by a Comet assay system (Trevigen, Inc, Gaithersburg, MD, USA) following the manufacturer's recommendations and as described earlier.^45^ The extent of DNA damage was calculated as percent DNA in the tail using OpenComet (v1.3), a plugin for the image processing program ImageJ. The study was repeated independently three times. The data are displayed as a box and whisker diagram showing median and middle quartiles with whiskers at the min and max.

### Immunofluorescence

Control and treated cells were immunostained with indicated antibodies as described.^45^ The TIF assay based on the co-localization detection of DNA damage markers (*γ*H2AX) and a telomeric protein (TRF1 or TRF2) was performed as described earlier.^46^ Detection of APBs (by double staining of telomeric proteins, such as TRF2, and a PML nuclear body-associated protein, such as PML) was performed as described.^47^ Stained cells were examined on a Zeiss Axiovert 200 M microscope and analysed by AxioVision 4.6 software (Carl Zeiss, Tokyo, Japan). At least, 200 cells (on duplicate slides) were evaluated for each treatment condition for co-localization foci counting. Three independent experiments were performed. Images were quantified by ImageJ software (National Institute of Health, Bethesda, MD, USA).

### Western blotting

Cells were lysed with radio-immunoprecipitation assay buffer (Thermo Scientific, Cambridge, UK) containing complete protease inhibitor cocktail (Roche Applied Science, Mannheim, Germany). Western blotting with indicated antibodies was performed as described earlier.^45^ Densitometric quantitation of the representative immunoblots was carried out using the ImageJ software (National Institute of Health). All experiments were performed in triplicate and at least three times.

### RNA extraction and real-time qRT-PCR

Total RNA was isolated from cells using the RNeasy mini kit (Qiagen). Equal amounts of RNA were used for reverse transcription following the protocol of QuantiTect Rev. Transcription Kit (Qiagen). The real-time qRT-PCR was performed on the Eco™ real time system (Illumina, San Diego, CA, USA) using SYBR® Select Master Mix (Applied Biosystems, Foster, CA, USA). Primers sets were listed in Supplementary Table 1. More details were given in Supplementary Information.

### Statistical analysis

The data from three or more experiments were expressed as mean±standard deviation. Two-tailed Student's *t*-test was used to compare control and treated groups. Statistical significance was defined as significant (*P-value≤0.05), very significant (**P-value≤0.01) and highly significant (***P-value≤0.001).

### Computational methods

#### Docking of Wi-A with Myc-Max complex and Mad-Max complex

Structures of Myc-Max and Mad-Max protein complexes with bound DNA (PDB IDs: 1NKP and 1NLW)^36^ were prepared using Protein Prep Wizard of Schrödinger software (Epik v3.3; Impact v6.8; Prime v4.1).^48^ The SiteMap package of the Schrödinger software was used to identify possible ligand binding sites at Myc-Max and Mad-Max complexes.^49^ Glide version 6.8 package of Schrödinger software was used to generate the grid at the identified ligand binding site using default parameters.^50^

Structure of Wi-A ligand (PubChem CID: 265237) was prepared using the LigPrep version 3.5 module of Schrödinger software. Glide version 6.8 was used to dock Wi-A at the identified binding site of protein complexes. Docked poses of Wi-A were superimposed over DNA-bound protein structures to serve two purposes: first, to check whether or not Wi-A was interfering with bound DNA, and second to generate DNA-ligand-protein complexes. These DNA-ligand-protein complexes were later used to study the effect of Wi-A binding on DNA binding ability of Myc-Max and Mad-Max proteins. Docking pose visualization, superimposition of protein structures and image generation was done using the Maestro version 10.3 Interface of the Schrödinger software. High ambiguity-driven protein-protein DOCKing (HADDOCK) 2.2 server^51^ was used to dock protein complexes with DNA.

#### Molecular dynamics simulations

The GPU-accelerated Amber Molecular Dynamics suite with Amber ff12SB protein force field was used to perform all atoms explicit molecular dynamics simulations of protein-ligand and protein-DNA-ligand complexes.^52^ Complex molecules were solvated with TIP3P water model in a cubic periodic boundary box and systems were neutralized using appropriate number of counter ions. The distance between box wall and protein complex was set to 10 Å. Neutralized systems were then minimized, slowly and gradually heated up to 300 K temperature, and equilibrated until the pressure and energies of systems were stabilized. Finally, equilibrated systems were used to run 50 ns long molecular dynamics simulations. All simulation studies were performed on DELL T3610 workstation with 16 GB DDR RAM and NVIDIA GeForce GTX TITAN Black Graphics Card.

#### Analysis of molecular docking and dynamics simulations

Visual Molecular Dynamics (VMD) version 1.9.2 was used to calculate RMSD and hydrogen bond dynamics.^53^ COCOMAPS was used to calculate the total interacting area/interface between DNA and proteins before and after the Wi-A binding.^54^, ^55^ Residue numbering of all the proteins discussed in this manuscript is according to PDB file.

## Figures and Tables

**Table 1:** Docking score of Wi-A after binding with proteins at DNA binding site, HADDOCK score between protein and DNA, and change in the binding interface between proteins and DNA after the binding of Wi-A

	**Myc-Max-DNA complex**		**Mad-Max-DNA complex**	
Docking score	−5.47 kcal/mol		−5.86 kcal/mol	
HADDOCK score	−190.6		−275.1	
Binding interface area	Ligand unbound	Ligand bound	Ligand unbound	Ligand bound
	1465.5 Å^2^	1724 Å^2^	1407 Å^2^	2966.4 Å^2^
