# Supplementary material for: Withaferin-A kills cancer cells with and without telomerase: chemical, computational and experimental evidences
Source: Cell Death Dis. 2017 Apr 20;8(4):e2755–. doi: 10.1038/cddis.2017.33 (PMC5477593; doi:10.1038/cddis.2017.33)
Supplement: Supplementary Information [file cddis201733x1.docx]

**Materials and methods**

**Luciferase reporter assay**

For wild type p53 promoter activity measurement, cells were co-transfected with 1 μg of luciferase constructs (PG13-luc, Addgene plasmid # 16442) and 100 ng of control oligonucleotide (pRL-TK) using Lipofectamine 2000 (Invitrogen, Carlsbad, CA, USA). After 24-48 h, luciferase activity was measured using a dual luciferase reporter assay system (Promega, Madison, WI, USA) following the manufacturer’s protocol. For NBN luciferase reporter assay, cells were grown in white 96-well plates, 20 ng of RenSP luciferase reporter plasmid and Cypridina TK control plasmid were transfected using XtremeGENE HP (Roche, Basel, Switzerland) per well. Cells were incubated with transfection mix for 24 h followed by treatment with Wi-A. After another 24 h, luciferase activity was measured using the LightSwitch Daul Assay System (SwitchGear Genomics) following the manufacturer’s protocol. The Cignal Myc Reporter kit (Qiagen) is used to monitor the activity of Myc-regulated signal transduction pathways in control and Wi-A treated cells. The Myc-responsive luciferase construct encodes the firefly luciferase reporter gene under the control of a minimal CMV promoter and tandem repeats of the E-box sequence. A firefly luciferase reporter construct without any transcriptional response elements served as the negative control. A constitutively active CMV promoter driven firefly luciferase reporter construct served as the positive control. A *Renilla* construct encoding the *Renilla* luciferase reporter gene under the control of a CMV promoter was used as an internal control to normalize transfection efficiencies. Cells were co-transfected with the firefly and *Renilla* luciferase reporters using X-tremeGENE HP DNA transfection reagents (Roche) following the manufacturer’s protocol. The cells were subsequently treated with Wi-A for 24 h before harvest. Light production related to luciferase expression was measured in a luminometer (infinite M200 PRO, TECAN, Switzerland) using the dual Luciferase kit (Promega). Experiments were carried out in triplicate and repeated at least three times. Data were represented with respect to the control that were set as 100.

**Telomerase activity detection**

Telomerase activity was determined with a PCR-based telomeric repeat amplification protocol (TRAP) enzyme-linked immunosorbent assay (ELISA) kit (Roche, Mannheim, Germany) following the manufacturer's protocol. In brief, cells were collected 24/48 h after Wi-A treatment, washed three times with cold PBS, homogenized in 200 μl cell lysis buffer, and incubated on ice for 30 min. For the TRAP reaction, 3 μg of cell extract was added to 25 μl of reaction mixture, and sterile water was added to a final volume of 50 μl. PCR was then performed as follows: primer elongation (30 min, 25°C), telomerase inactivation (5 min, 94°C), product amplification for 30 cycles (94°C for 30 sec, 50°C for 30 sec, and 72°C for 90 sec) and then balance (10 min at 72°C). A total of 5 μl of PCR products was added to a streptavidin-coated 96-well plate and hybridized to a digoxigenin (DIG)-labeled telomeric repeat-specific detection probe. The immobilized PCR products were detected with peroxidise-conjugated anti-DIG antibody. After addition of the stop reagent, the plate was assessed with a plate reader at a wavelength of 450 nm within 30 min.

**RNA extraction and real-time qRT-PCR.**

Total RNA was prepared from control and Wi-A treated cells using RNeasy mini kit (Qiagen, Standford Valencia, CA, USA). The concentration and purity of RNA were determined by ultraviolet spectrophotometry (A_260_/A_280_ >1.9) using NanoDrop ND-1000 (Nanodrop Technologies, Wilmington, DE, USA). 1 µg of RNA were used for reverse transcription with random hexamers, following the protocol of QuantiTect Rev. Transcription Kit (Qiagen). Real-time PCR reaction was carried out in triplicate on Eco^TM^ Real-Time PCR System (Illimina, San Diego, CA, USA) in 10 μl mixture containing cDNA, indicated primers and SYBR® Select Master Mix (Applied Biosystems, Foster, CA, USA). The qPCR oligonucleotide primers sets and efficacies were provided in Supplementary Table 2. The qPCR condition was 50°C for 2 min, 95°C for 10 min followed by 40 cycles of denaturing at 95°C for 15 sec, and annealing at 60°C for 1 min. Melt curve was then generated to assess specification of the PCR amplification. Reaction efficiency was evaluated by performing a 10-fold dilution series experiment and calculated as E = 10^(-1/slope)^ – 1. Geometric mean of housekeeping gene 18S was used as an internal control to normalize the variability in expression levels. The results were analyzed and expressed as relative expression of threshold cycle value, which was then converted to x-fold changes using 2 ^− △△Ct^ method.^1^

**Telomere length measurement by quantitative PCR**

Total genomic DNA was purified using Wizard® Genomic DNA Purification Kit (Promega), following the manufacturer’s instructions. The DNA concentration (ng/µL) of each sample was measured using NanoDrop ND-1000 (Nanodrop Technologies, Wilmington, DE, USA) and aliquots diluted to 10 ng/µL with TE buffer. Samples were stored at -30°C. The assessment of relative telomere length was performed using a real-time quantitative PCR method previously described^2^ with some modifications in primer design and concentration. The primer sequences of telomere (telo) and single copy gene (36B4)^3^ were indicated in Supplementary Table 2. Primer concentrations were optimized for each batch in accordance with Power SYBR® Green PCR Master Mix guidelines (Applied Biosystems). For telomere reaction, 10 µl PCR reactions were set-up and contained the following reactants: 10 ng gDNA; 5 µl Power SYBR® Green PCR Master Mix (Applied Biosystems); 500 nM forward and reverse telo primers and 2 µl double-distilled water. Thermal cycling was performed on an Eco™ real time system (Illumina, San Diego, CA USA), with an initial incubation step at 95°C for 10 min, followed by 35 cycles of 95°C for 15 s and 54°C for 2 min. For 36B4 reaction, 10 µl PCR reactions were set-up and contained the following reactants: 10 ng gDNA; 5 µl Power SYBR® Green PCR Master Mix (Applied Biosystems); 500 nM forward and reverse 36B4 primers and 2 µl double-distilled water. Thermal cycling was performed with an initial incubation step at 95°C for 10 min, followed by 30 cycles of 95°C for 15 s and 58°C for 1 min. Reactions were run in triplicate for each sample. No template control reactions were carried out for each primer set during every qPCR run to ensure no contamination. Standard curves were created using the pool of gDNA samples (Control and Wi-A treated TEP and ALT cells) for both telomeres and 36B4 to ensure consistent rates of amplification over wide ranges of DNA concentration. Two-fold serial dilutions were applied, starting at a concentration of 80 ng/µl down to 1.25 ng/µl, with 7 different concentrations in total (Supplementary Figure S2A). The amplification efficiency of telomere and 36B4 were 0.98 and 0.99, respectively. All samples fell within the concentration range generated by the standard curve. The cycle threshold (C_t_) of telomere (T) and 36B4 (S) were used to calculate the relative telomere length with the calculation T/S (2 ^− △△Ct^).

**Supplementary Table 1.** SV40-immortalized human fibroblast lines

| Cell line name | Abbreviation | Telomere maintenance mechanism | Reference |
| --- | --- | --- | --- |
| JFCF-6/T.1J/6B | JFCF-6B | Telomerase | (4) |
| JFCF-6/T.1J/6G | JFCF-6G | Telomerase | (5) |
| JFCF-6/T.1L | JFCF-1L | ALT | (6) |
| JFCF-6/T.1J/1-4D | JFCF-4D | ALT | (7) |

**Supplementary Table 2.** Sequence and efficiency of primers used in real-time qRT-PCR.

| **Gene (human)** | **Sequence (5'-3')** | **Efficiency** |
| --- | --- | --- |
| NBS1 forward | AAGCAGATACATGGGATTTGAGTG | 99.8% |
| NBS1 reverse | TGGAGACTTTGATTTGATTTCTTTTGGC |  |
| RAD50 forward | GAATTATCCACTGAAGTTCAGT | 97.6% |
| RAD50 reverse | TCCAAAGGGCTTACCTGCTC |  |
| MRE11 forward | GCCTTCCCGAAATGTCACTA | 97.1% |
| MRE11 reverse | TTCAAAATCAACCCCTTTCG |  |
| 18s forward | CAGGGTTCGATTCCGTAGAG | 102.1% |
| 18s reverse | CCTCCAGTGGATCCTCGTTA |  |
| telomere forward | CGGTTTGTTTGGGTTTGGGTTTGGGTTTGGGTTTGGGTT | 98.2% |
| telomere reverse | GGCTTGCCTTACCCTTACCCTTACCCTTACCCTTACCCT |  |
| 36B4 forward | CAGCAAGTGGGAAGGTGTAATCC | 99.0% |
| 36B4 reverse | CCCATTCTATCATCAACGGGTACAA |  |

**A**


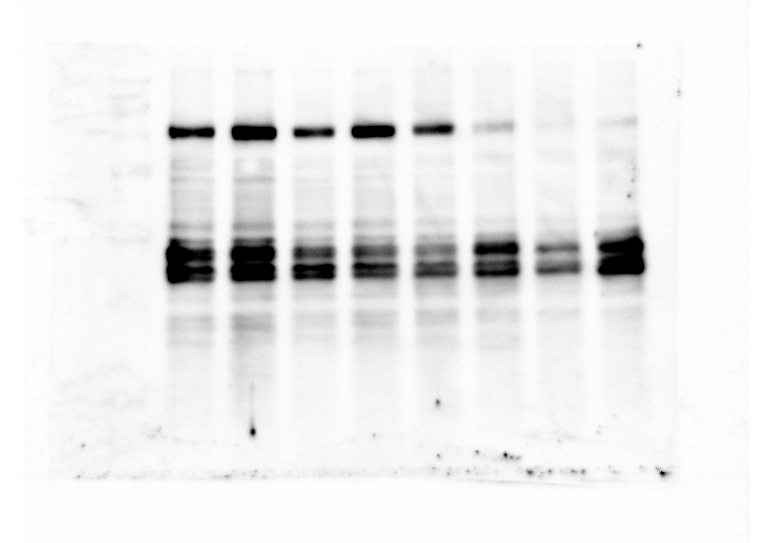

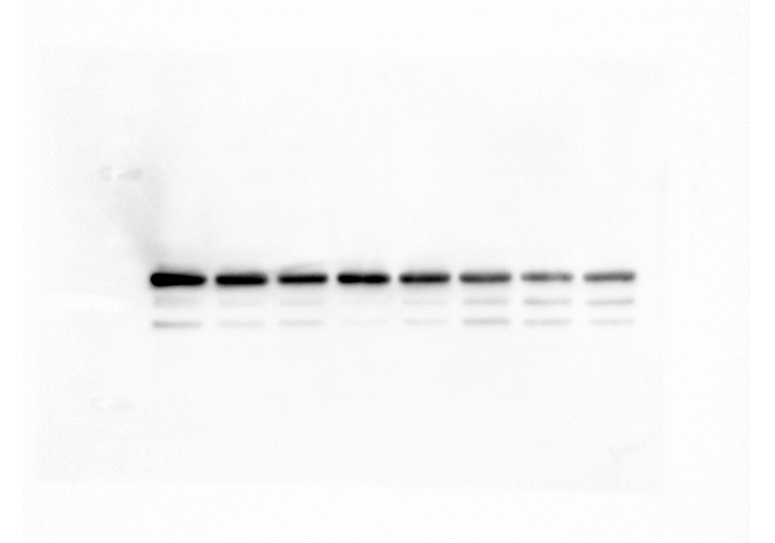

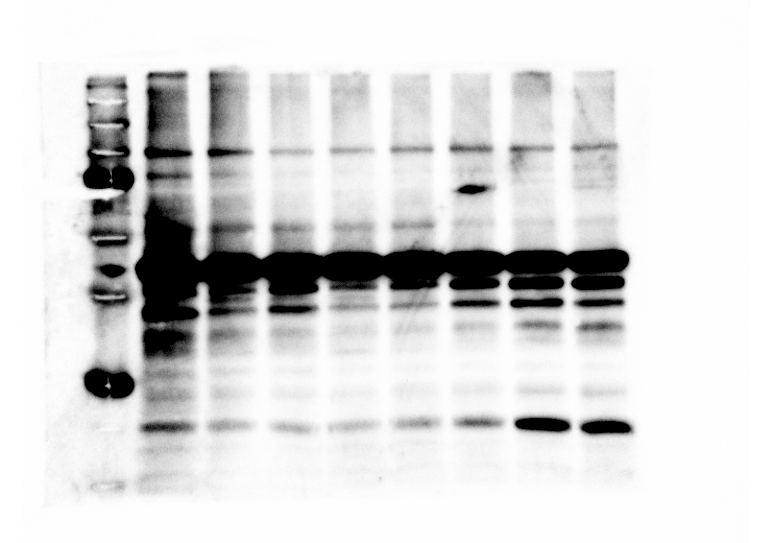

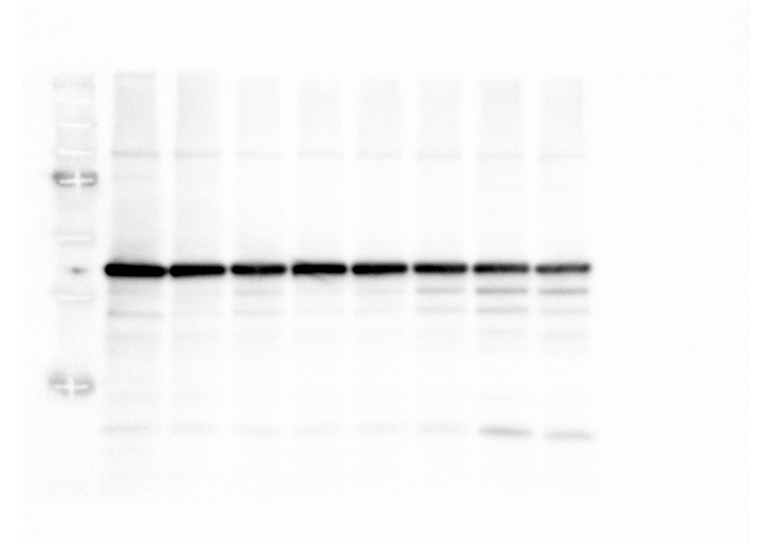


**Control**

**Wi-A**

**MCF7 (TEP)**

**Control**

**Wi-A**

**U2OS (ALT)**

***NBS1***

***β-actin***

**100**

**50**

**(KDa)**

**B**

**Supplementary Figure S1.**

(A) Cell viability assays showing higher cytotoxicity of Wi-A to tumor derived ALT (U2OS) cells than TEP (MCF7) cells. (B) Western blot analysis of control and Wi-A treated TEP (MCF7) and ALT (U2OS) cells showing decrease in NBS1 in Wi-A treated ALT, but not TEP, cells.

**A**

**B**

**Supplementary Figure S2.**

(A) Standard curves were generated to determine the consistency of the qPCR reactions over a wide range of concentrations. Diamonds represent telomeres and squares represent the reference gene 36B4. (B) qPCR-based telomere length measurement showing no effect of Wi-A on telomere length in both ALT and TEP cells.


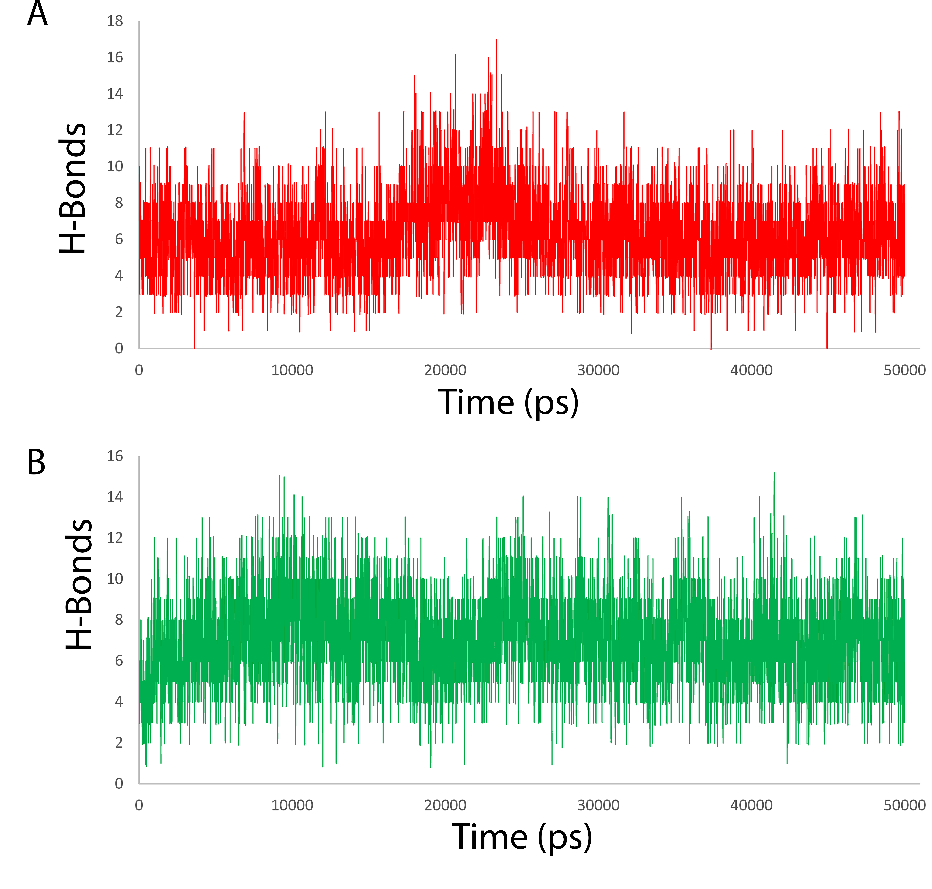


**B**

**A**

**Supplementary Figure S3.**

Change in number of hydrogen bonds between DNA and protein after the binding of Wi-A with Myc-Max (Frame A) and Mad-Max (Frame B) proteins. Overall, the number of hydrogen bonds increased after the binding of Wi-A, with the increase being highest when Wi-A is bound to Mad-Max protein.

1. Livak KJ, Schmittgen TD. Analysis of relative gene expression data using real-time quantitative PCR and the 2(-Delta Delta C(T)) Method. *Methods* 2001; **25:** 402-408.

2. Cawthon RM. Telomere length measurement by a novel monochrome multiplex quantitative PCR method. *Nucleic Acids Res* 2009; **37:** e21.

3. O'Callaghan NJ, Fenech M. A quantitative PCR method for measuring absolute telomere length. *Biol Proced Online* 2011; **13:** 3.

4. Jiang WQ, Zhong ZH, Nguyen A, Henson JD, Toouli CD, Braithwaite AW*, et al.* Induction of alternative lengthening of telomeres-associated PML bodies by p53/p21 requires HP1 proteins. *J Cell Biol* 2009; **185:** 797-810.

5. Kaul Z, Cesare AJ, Huschtscha LI, Neumann AA, Reddel RR. Five dysfunctional telomeres predict onset of senescence in human cells. *EMBO Rep* 2012; **13:** 52-59.

6. Lovejoy CA, Li W, Reisenweber S, Thongthip S, Bruno J, de Lange T*, et al.* Loss of ATRX, genome instability, and an altered DNA damage response are hallmarks of the alternative lengthening of telomeres pathway. *PLoS Genet* 2012; **8:** e1002772.

7. Yuan J, Yang BM, Zhong ZH, Shats I, Milyavsky M, Rotter V*, et al.* Upregulation of survivin during immortalization of nontransformed human fibroblasts transduced with telomerase reverse transcriptase. *Oncogene* 2009; **28:** 2678-2689.
